# Editorial: Cardiac issues in adults with mucopolysaccharidosis

**DOI:** 10.3389/fcvm.2022.1016386

**Published:** 2022-08-23

**Authors:** Karolina M. Stepien, Elizabeth A. Braunlin

**Affiliations:** ^1^Inherited Metabolic Diseases Department, Salford Royal NHS Foundation Trust, Manchester, United Kingdom; ^2^Division of Diabetes, Endocrinology and Gastroenterology, University of Manchester, Manchester, United Kingdom; ^3^Department of Pediatrics, University of Minnesota, Minneapolis, MN, United States

**Keywords:** adult MPS, cardiac, newborn screening, guidelines, transition, biomarkers, cardiac surgery, enzyme replacement therapy

The focus of this issue of the Frontiers in Cardiovascular Medicine is on cardiac manifestations in adults affected with Mucopolysaccharidoses (MPS). Conceptual advances that have had practical applications will be highlighted in this issue. Although rare, MPS are a global problem and represent life-limiting or threatening disorders that can have a significant impact on patients' wellbeing. Experts have been chosen from around the world, whose experience reflect the forefront in medical genetics/metabolic medicine devoted to progress in the MPS.

Over the past few decades, we have witnessed remarkable progress in our understanding of MPS; a heterogenous group of lysosomal storage diseases characterized by a disruption in the degradation of glycosaminoglycans (GAGs) in the lysosome. The extent of changes in the arterial vessel in MPS has not been well-defined, but it has been speculated that their endothelial dysfunction/dysregulation may be primed by the accumulation of GAGs, such as chondroitin 6-sulfate in Morquio syndrome, developing atherosclerotic lesions and resulting in increased carotid intima media thickness (1). The study by Montavon et al. evaluates the role of biomarkers in the etiology of cardiovascular disease in Morquio syndrome. Two non-specific biomarkers cathepsin C and elastin are the first biomarkers of extracellular matrix ever been evaluated in MPS disorders.

The lack of diagnostic and therapeutic biomarkers is a significant gap in the knowledge of cardiac disease in adult MPS. A second article offers risk stratification prior to cardiac valve surgery to reduce the peri-operative mortality and improve the recovery after the procedure (Cross et al.). Given the heterogenous character of the MPSs and their rarity, the collaborative work among metabolic and genetic centers around the world is necessary. To date the de-centralized nature of the US health care system has prevented an accurate estimate of the incidence of MPS. Going forward, newborn screening in the US may provide that information. Integration of genomic data into the electronic medical record is becoming increasingly available and, if integrated across the US health care systems, could ultimately advance understanding of the effects of as-yet unknown factors that influence the clinical course of MPS. The call is to pharmaceutical industry for launching and funding the research in metabolomics, proteomics and lipidomics. The highly sensitive and specific cardiovascular biomarkers are essential and could potentially improve the long-term clinical outcomes of adults with MPSs.

It is evident that, historically, GAG degradation results in prominent storage of undegraded GAGs in heart structures, which results in tissue damage through the activation of cell proteases (2). Heart valve disease and conduction abnormalities, often seen in adult MPS, may result in increased mortality (3, 4). It does not however apply to all MPS patients, but the factors determining the age of onset or specific vulnerability remain undefined. Studies that have attempted to correlate clinical phenotype with the genotype have repeatedly pointed to the lack of perfect concordance.

Better understanding of the cardiovascular disease pathogenesis in MPS is key to implementing better diagnostic modalities in routine clinical practice. Lack of clear guidelines on cardiopulmonary investigations, their frequency and interpretation remain an unmet need and are addressed in a separate article (Stepien and Braunlin). Electrocardiogram, echocardiogram and 24-h electrocardiogram are available in every hospital around the world, but due to limited disease awareness, clinicians may not be cognizant that adult MPS patients still require close cardiac surveillance in the pre-symptomatic stage of the disease due to the progressive nature of the disease. As noted by de Oliviera Poswar et al. available enzyme replacement therapy is not able to reverse many of the cardiac issues in the MPSs so the new upcoming therapies with different pharmacodynamics and pharmacokinetics, would preferably enhance the GAGs degradation and prevent their deposition in heart valves and conduction pathway.

In a final paper of the series, Stepien et al. describe common non-cardiac clinical complications in adult MPS patients irrespective of the therapy. Some presentations may be MPS-type specific; some are seen only in adulthood or are exacerbated by advanced age. The pathophysiology of some complications such as refractory iron deficiency anemia or gastrointestinal dysfunction warrant further research.

As evident from the articles in this issue, the MPS disorders continue to generate new insights into basic molecular processes that inform us of potential pathophysiologic mechanisms and therapeutic strategies. Further advances aimed at reducing mortality and improving patients' quality of lives can be expected, as the ongoing diagnostic and therapeutic development is sustained by the expanding number of researchers in the metabolic medicine field.

## Author contributions

KS and EB: concept and design. All authors read and approved the final version of the manuscript.

## Conflict of interest

The authors declare that the research was conducted in the absence of any commercial or financial relationships that could be construed as a potential conflict of interest.

## Publisher's note

All claims expressed in this article are solely those of the authors and do not necessarily represent those of their affiliated organizations, or those of the publisher, the editors and the reviewers. Any product that may be evaluated in this article, or claim that may be made by its manufacturer, is not guaranteed or endorsed by the publisher.
